# ‘From scared to prepared’: targeted structured induction training during the transition from medical school to foundation doctor

**DOI:** 10.1007/s40037-015-0168-x

**Published:** 2015-04-14

**Authors:** Natalie S. Blencowe, Clare Van Hamel, Rob Bethune, Rebecca Aspinall

**Affiliations:** 1 Division of Surgery, Head and Neck, University Hospitals Bristol NHS Foundation Trust, Bristol, UK; 2 Foundation School, Severn Deanery, Bristol, UK; 3 Sir Humphry Davy Department of Anaesthesia, University Hospitals Bristol NHS Foundation Trust, Marlborough Street, Bristol, UK

**Keywords:** Induction, Preparing for practice, Medical students, Foundation doctors

## Abstract

The risks to patients at August handover time are well known, yet there is no national consensus on the best way to deliver induction programmes for Foundation Year One (F1). The aim of this study was to design, deliver and assess a targeted structured induction programme for new F1 doctors. The induction training programme was designed using educational models of topic analysis informed by results of a survey of F1s and medical students, and the F1 curriculum. Data regarding serious untoward incidents and self-reported preparedness were collected between 2008 and 2010, and rates were compared between those receiving optional (2008) and compulsory (2009 and 2010) training. By delivering targeted education and spending time with the outgoing F1 doctors, 97 % of our new doctors felt adequately prepared for practice. The incidence of self-reported mistakes made by F1s in the first 4 months of their practice fell by 45 % and serious untoward incidents also decreased. Targeted structured induction training addresses final-year medical students’ concerns about their preparedness for practice as junior doctors, and improves patient safety. This study supports the General Medical Council recommendation that targeted structured induction training should be mandatory for all new doctors.

## Background

‘Don’t be ill in August’ is a statement familiar to most doctors and increasingly the media, as patients admitted at the beginning of August (when newly qualified doctors start work in NHS hospitals in the UK) have a higher death rate compared with the previous week [1]. This supports existing findings from a multi-centre study conducted in the US [2]. The General Medical Council’s ‘Tomorrow’s Doctors’ document states that ‘students must be properly prepared for their first day as a Pre Registration House Officer’ [3]. However, in a 2005 national survey, 41.5 % of new doctors felt inadequately prepared with respondents commenting that there was ‘not enough emphasis on real life situations’ and ‘not enough time shadowing’ [4]. Additionally, more than a third of F1 doctors feel they are asked to perform tasks beyond their capabilities at some stage during the pre-registration year [5]. Feeling overwhelmed and underprepared is thought to increase stress levels in junior doctors and can affect their quality of life [6], with just 30 % of new doctors having a strong desire to practice medicine on completing their first Foundation year [5].

Newly qualified F1 doctors starting work in the UK usually undertake corporate induction, which involves mandatory training (such as fire safety, manual handling, basic life support and antibiotic guidelines) and varies according to local hospital policy. This does not, however, involve any specific training to prepare them for practice in the workplace. During two locally delivered teaching sessions on ‘learning from each other’s mistakes’ in 2007, it became apparent that F1s had not felt prepared to start working as professional doctors and that this might be addressed through targeted education. Subsequently, the ‘From Scared to Prepared’ course was designed.

## Course design

In 2008 an email survey of current F1 doctors and final-year medical students was conducted. Medical students were asked what they were most apprehensive about prior to starting work. F1 doctors were asked what they wished they had been taught before commencing their post. Responses to these questions defined the aim of the course as ‘developing clinical knowledge, skills and attitudes of a level appropriate for an F1, in order to achieve a safe standard of practice’. In keeping with previous results from national surveys, ‘surviving the night shift’ and ‘managing critically unwell patients’ were the most common responses amongst those surveyed. Using educational models of topic analysis, the one-day ‘From Scared to Prepared’ course was designed using this information and was delivered in late July according to pre-specified objectives. Each session contained specific objectives and the content was chosen to provide information that was considered to be most important in starting as an F1 doctor. Individual sessions included ‘top ten bleeps’ (e.g. ‘the hypotensive patient’, ‘the patient with chest pain’ etc.), blood gas/X-ray/biochemistry interpretation, documentation, handover, and fluid and drug prescribing.

## Course delivery

A facilitator and a small faculty of doctors at the end of their F1 year delivered all aspects of the ‘From Scared to Prepared’ course. It was felt, having qualified most recently, these doctors would understand the new F1 cohort’s fears and concerns and be able to recognize and focus on the most important areas in preparing for starting work. The teaching was interactive and included group-based exercises, role play and written tasks, reflecting key areas important for safe practice as well as advice which may not have been immediately accessible during undergraduate training. Other sessions included corporate induction elements, training at the Bristol Medical Simulation Centre focusing on the management of acutely ill patients, and an e-learning programme for safe prescribing. Additionally, two days were spent shadowing the outgoing F1 doctor whom they were replacing. F1s then started work on the wards on the first Wednesday in August with prior knowledge of the patients they were responsible for, whilst other senior doctors attended mandatory induction.

In 2008, all doctors about to start F1 jobs at University Hospitals Bristol NHS Foundation Trust were offered the five-day targeted structured induction training. Participation was voluntary but unpaid and the objective and nature of the course was explained in a letter sent by the Foundation Programme Director. The course was attended by 29 of 39 new F1s. In 2009, based on feedback and interest from trust management, the course became a compulsory part of the induction process. All 39 new F1s were paid a salary to attend. In 2010, the course was delivered to all new F1 doctors in Severn and Peninsula deaneries, all of whom were paid a salary.

## Course evaluation

The F1s were asked to complete a confidential questionnaire after four months of work. This asked about their preparedness for starting work as a doctor, and critical incidents with which they had been involved. In 2008, 83 % (24 of 29) of F1s who attended the course felt prepared for their first month as a doctor compared with 1 of 10 of those not attending. In 2009, all 39 new F1s attended the course and of these, 38 (97 %) felt prepared on their first day as doctors.

Critical incidents were classified according to the nature of the error as well as severity, and a Delphi survey was used to rate the severity of incidents by asking ten NHS consultants to weigh adverse incidents on a scale between 1 and 100. Delphi weightings of adverse incidents allocated ‘patient death’ a score of 100, ‘permanent patient harm or intensive care admission’ 90, ‘transient patient harm’ 75, and ‘no patient harm’ 25.

In 2008, there were 96 self-reported incidents, five of which resulted in permanent patient harm (Delphi weighting score 3020). In 2009, there were 52 self-reported incidents, one of which resulted in permanent patient harm (Delphi weighting score 1665). This represents a 45 % reduction in critical incidents from 2008 to 2009. In 2008, the relative risk of a clinical incident was higher amongst those F1s who had not attended the induction programme (Table 1).

**Table 1:** Self-reported mistakes and course attendance in 2008

	Patient harm		No patient harm	
	Did not attend course	Attended course	Did not attend course	Attended course
Total mistakes	4	1	19	69
Relative risk	0.44	0.03	2.11	2.3

## Discussion

Mandatory trust induction is delivered to all new employees and focuses on elements prescribed by national insurance policies; however, the frequency and severity of mistakes that new doctors make are not addressed. Trusts have implemented various strategies, from one-day corporate induction to one or more days of handover and shadowing. Before the national scheme was implemented in 2012, only one other Deanery (East of England) provided a formalized, paid early start for F1s; others were voluntary, unpaid and ad hoc. The introduction of Modernizing Medical Careers has seen the development of a competency-based curriculum for foundation doctors, with workplace-based assessments forming the backbone of formative and summative assessment [7]. The use of these tools has also extended into the assessment process of speciality trainees [8], yet they are not used in the medical school setting. Medical schools must achieve a balance between producing doctors with adequate knowledge who can also deliver patient-centred care, which may necessitate modernization of the undergraduate curriculum.

Although this study reports improved preparedness amongst F1 doctors and improved patient safety it has several limitations. The cohort of doctors who chose not to attend in 2008 are a self-selected population and may have made these mistakes even if they had attended the course. Additionally, anonymized self-reporting relies on these doctors remembering all their mistakes retrospectively, which may result in an under-reporting of critical incidents. Although serious incidents were confirmed using the hospital’s critical incident reporting system, minor mistakes were not captured and therefore could not be validated. Finally, this study looked only at events during the first four months of F1 practice and whether a reduction was sustained throughout the year cannot be determined.

The risks to patients at August handover time are well known [1], yet until recently there was no national consensus on the best way to deliver induction programmes for F1 doctors, resulting in considerable variation between hospital trusts. As of August 2012, a four day long induction period was made mandatory by NHS England, but the specifics of the induction were not mandated [9]. The ‘From Scared to Prepared’ course has now been fully funded by the South West Strategic Health Authority, allowing all new F1s in the Peninsula and Severn Deaneries to start their paid employment one week before the outgoing F1s leave for F2 posts. It is proposed that this educational intervention will improve patient safety with additional cost saving benefits.
